# Microencapsulated Quercetin and *Bifidobacterium animalis* Independently Preserve Jejunal Enteric Neurons During Colorectal Carcinogenesis

**DOI:** 10.1111/nmo.70190

**Published:** 2025-10-29

**Authors:** Lucas Casagrande, Carla Cristina de Oliveira Bernardo, Sabrina Silva Sestak, Maysa Pacheco Alvarez da Silva, Marcos Yudi Nagaoka Godoy, Jean‐Pierre Timmermans, Cesar Agostinho Ferreira, Tânia Cristina Alexandrino Becker, Erick Guilherme Stoppa, Waldiceu Aparecido Verri, José Ricardo de Arruma Miranda, Juliana Vanessa Colombo Martins Perles, Jacqueline Nelisis Zanoni

**Affiliations:** ^1^ Pharmaceutical Sciences Graduate Program University of Maringá Paraná Brazil; ^2^ Laboratory of Cell Biology & Histology University of Antwerp Antwerp Belgium; ^3^ Health Sciences Graduate Program State University of Maringá Paraná Brazil; ^4^ São Paulo State University São Paulo Brazil; ^5^ State University of Londrina Paraná Brazil

**Keywords:** colorectal carcinogenesis, enteric neuronal density, intestinal transit, microencapsulated quercetin, probiotic

## Abstract

**Background:**

Colorectal cancer (CRC) is a leading cause of cancer‐related deaths, significantly disrupting enteric neurotransmission within the colon. While the effects of CRC on the enteric nervous system (ENS) of the colon are well documented, its impact on the small intestine remains underexplored. This study aims to investigate the influence of colorectal carcinogenesis on the small intestine's ENS and evaluate the individual neuroprotective effects of microencapsulated quercetin and *Bifidobacterium animalis* .

**Methods:**

Wistar rats were subjected to chemically induced colorectal carcinogenesis, followed by 14 weeks of treatment with microencapsulated quercetin and *B. animalis* . Gastrointestinal transit times were assessed, and colonic and jejunal samples underwent histopathological and immunohistochemical analyses to evaluate neuronal markers (HuC/D, nNOS, VIP). Cholinergic neurons were not directly assessed.

**Results:**

Aberrant crypt foci confirmed the effectiveness of the colorectal carcinogenesis induction model. The mean gastric emptying time (MGET) was notably shorter in the *B. animalis* ‐treated group. Colorectal carcinogenesis significantly reduced the density and size of HuC/D+ neurons in the myenteric and submucosal plexuses of the jejunum. Treatments with either microencapsulated quercetin or *B. animalis* significantly enhanced neuronal density and size in the jejunum and improved nitrergic neuronal density (nNOS‐IR). Additionally, VIPergic neuron density increased in the submucosal plexus, and varicosity size increased in the myenteric plexus in the CR group; treatments reduced this varicosity size.

**Conclusion:**

This study provides the first evidence that colorectal carcinogenesis damages jejunal neurons. Treatments with microencapsulated quercetin or *B. animalis* independently preserved neuronal density and modulated gastrointestinal function. However, their combined administration did not enhance these effects, highlighting the need for further research into therapeutic interventions for preserving ENS integrity during colorectal carcinogenesis.

## Graphical abstract

Our study explores the impact of colorectal carcinogenesis on jejunal neurons of the enteric nervous system (ENS), revealing significant neuronal damage. We demonstrated that treatments with microencapsulated quercetin and *Bifidobacterium animalis* protect enteric neuronal density, and this is reflected in improved gastrointestinal transit times. Our findings suggest promising therapeutic potential to preserve the integrity of the ENS and gastrointestinal function during colorectal carcinogenesis.

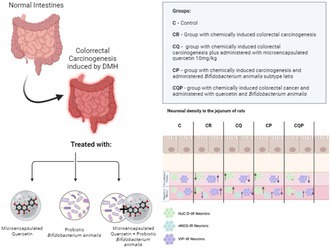

**Summary**

- Colorectal carcinogenesis significantly damages enteric neurons in the jejunum, a novel finding that expands the known impact of CRC beyond the colon.
- We demonstrated that treatment with either microencapsulated quercetin or *Bifidobacterium animalis* preserved enteric neuronal density independently. Only *B. animalis* reduced gastric emptying time.
- These findings suggest the potential of individual treatments as adjunct therapies to preserve ENS integrity during colorectal carcinogenesis, while the combination of both agents did not enhance the neuroprotective effects.

## 1 Introduction

Colorectal cancer (CRC) is the third most common type of cancer worldwide, according to the International Agency for Research on Cancer (IARC), with the highest incidence rate. It is also the second deadliest type of cancer [1].

CRC is characterized by the presence of aberrant crypts (AC), which can progress to adenomas and eventually to carcinoma. AC is a preneoplastic lesion that is made up of crypts with larger and thicker epithelium than normal crypts, often grouped together in foci. Aberrant crypt foci (ACF) may exhibit hyperplasia or dysplasia, leading to the transformation of the mucosal epithelium into a hyperproliferative epithelium that loses organization and forms adenomas [2]. Adenomas grow and invade the submucosa and can become cancerous, with the potential to spread throughout the colon [3].

Although there is no literature on the effects of colorectal carcinogenesis on the enteric nervous system (ENS) of adjacent organs, such as the small intestine, it is known that structural alterations in the ENS commonly occur in the large intestine, resulting in a gradual reduction and destruction of nervous structures [4]. The importance of enteric neurons in CRC in the colon was reviewed by Schonkeren et al. in 2021 [5], focusing on the link between the ENS, inflammation, and CRC, as well as perineural invasion and neurotransmitters involved in CRC. The small intestine is known to function when isolated from the central nervous system (CNS), depending on the ENS to control its various movements, as well as the movement of fluid across the epithelium, local blood flow and the interactions with the endocrine and immune systems of the intestine [6]. Therefore, this study presents, for the first time, the effects of colorectal carcinogenesis on the ENS in the jejunum of rats induced with chemical colorectal carcinogenesis.

Quercetin is a flavonoid that has been widely studied for its antioxidant function. In addition to its antioxidant activity, this compound has anti‐inflammatory and antitumor effects, with the latter resulting from mechanisms such as the modification of important carcinogenesis signaling pathways and interactions with specific proteins and receptors [7]. To improve its pharmacokinetic aspects and reduce toxicity while using lower doses, we use microcapsules [8, 9].

Likewise, *Bifidobacterium* is an important part of the commensal microbiota, with several functions in homeostasis, including immunomodulatory, metabolic, and anti‐inflammatory roles. *Bifidobacterium* strains have protective and preventive effects on microbiota composition and may influence the epigenetic regulation of CRC [10]. Given that both quercetin and *Bifidobacterium* strains individually modulate gut microbiota and exert anti‐inflammatory effects, their combined administration has been proposed as a strategy to enhance these protective outcomes, potentially offering synergistic benefits [11]. We hypothesized that such effects, both from microencapsulated quercetin and *Bifidobacterium animalis* subtype lactis, would be able to preserve enteric neurons during colorectal carcinogenesis.

## 2 Materials and Methods

### 2.1 Experimental Design

All procedures described in this study are in accordance with the ethical principles of the Brazilian Society of Laboratory Animal Science. The experimental protocol (number: 1126010419) was approved by the Ethics Committee for the Use of Animals in Experiments at the State University of Maringá.

Forty adult male Wistar rats ( *Rattus norvegicus* ), from the central animal facility of the State University of Maringá, at 50 days of age, were transferred to the sectorial Vivarium of the Department of Morphological Sciences, maintained under controlled environmental conditions of temperature (24°C ± 2°C), a 12‐h light/12‐h dark cycle with lights on during the day, food (Nuvilab, Colombo, Paraná) and water ad libitum. At 53 days of age, following a 3‐day adaptation period to the new environment, the rats entered the experimental period that lasted for 16 weeks.

The animals were distributed into 5 groups with 8 rats each, as follows: Control (C), chemically induced colorectal carcinogenesis (CR), induced, and administered with microencapsulated quercetin 10 mg/kg, (CQ), induced and administered with *Bifidobacterium animalis* (Active Pharmaceutica, Pinhais, Brazil) subtype lactis 5 × 10^7^UFC (CP) and induced and receiving both treatments (CQP). The animals belonging to the CR, CQ, CP, and CQP groups were injected intraperitoneally with 1,2‐dimethylhydrazine (DMH) (Sigma‐Aldrich, St. Louis, USA), 40 mg/kg twice a week for two weeks [12], while those in group C received a sterile saline solution to minimize interference. Both the C and CR groups were gavaged with filtered water, which served as the vehicle for the treatments.

Microencapsulated quercetin was administered at a dose of 10 mg/kg by gavage daily [9]. The administration of the probiotic *Bifidobacterium animalis* subtype lactis was by gavage with a solution containing 5 × 10^7^UFC in the appropriate volume for the weight of each rat [10]. The animals were weighed every 2 days, and their individual dose was calculated.

### 2.2 Preparation of Quercetin Microcapsules

The preparation of quercetin microcapsules was performed according to the method described by Baracat et al. [13]. Aqueous dispersions of pectin and casein (8.34%, w/v; pH 8.0 ± 0.1) were made under solution. Quercetin (Acros, New Jersey, USA) was dispersed in deionized water under mechanical injection and distributed to the pectin dispersion (CP Kelco, Limeira, Brazil). Then, the casein dispersion (Kauffman & Co, Kehl, Germany) was added and the microcapsules were suspended slowly, dispersed in the pH 3.5 ± 0.1 by adding 1.0 M citric acid (Merck, Darmstadt, Germany). The microcapsules were stiffened by adding glutaraldehyde at 50 μL/g polymer (Sigma, St. Louis, USA). The microencapsulated quercetin was spray‐dried using a spray dryer (LabPlant, SD‐05).

The quantification of quercetin in the formulation was performed according to the quantification method of total flavonoids [14]. Samples were dispersed in 80% ethanol, mixed for 15 min, and centrifuged for 10 min at 3000 rpm. Aliquots of the supernatant were added in 2% aluminum chloride prepared in 80% ethanol. After 1 h of incubation at room temperature, absorbance was determined at 420 nm. This process was repeated in the same sample until the complete admission of quercetin to the formulation. The amount of quercetin was determined using the standard quercetin calibration curve at concentrations 6, 8, 12, 16, 20, 24, and 30 μg/mL of 80% ethanol. The % of quercetin incorporated, compared to the total quercetin presented in the formulation, reached 16.7% of the weight of the microcapsule loaded with quercetin.

### 2.3 Gastrointestinal Transit

Gastrointestinal motility was assessed using a non‐invasive technique known as Alternate Current Biosusceptometry (ACB), which allows real‐time detection of magnetic materials along the digestive tract. Magnetic monitoring was performed by measuring the intensity values recorded by the Mono‐ACB system positioned on the abdominal surface. After 90 days of the experimental period, the animals were handled gently by the neck, and the sensor was positioned on their gastric and cecum projection after ingesting solid magnetic meals. The stomach and cecum regions were determined through the anatomical references defined in previous studies [15, 16, 17]. This system enabled the monitoring of gastric emptying (GE) and orocecal transit (OCT).

The magnetic meal (Pellet 2 g) was composed of powder ferrite (0.5 g) and laboratory chow (1.5 g). The manganese microparticles (ferrite, MnFe2O4, 50–100 μm, Ferroxcube, El Paso, USA) supply an excellent magnetic response and do not need the previous magnetization. Furthermore, it is non‐absorbable and inert to the body [17].

After ingesting the solid magnetic meals, the GE and OCT were obtained by recording the signal intensity from subsequent measurements at 15‐min intervals for 6 h in the anatomical regions of the stomach and cecum, respectively [15, 18]. The following measures were performed at the described interval with the animals awake and manipulated by the neck.

The raw signals obtained in each anatomical region were analyzed by visual inspection and a statistical moment analysis. The first temporal statistical moment was defined as the average time point of the magnetic signal intensity curve, weighted by the signal amplitude at each time, and normalized by the total area under the curve (AUC). Therefore, we quantified two relevant parameters. The Mean Gastric Emptying Time (MGET) was defined as the time point (in minutes) representing the average residence time of the magnetic meal in the stomach, calculated from the descending gastric signal curve. The Mean Cecum Arrival Time (MCAT) was defined as the average time of arrival of the magnetic meal to the cecum, calculated from the ascending curve of magnetic signal intensity and based on the first temporal statistical moment [15]. These procedures follow the analytical protocol validated in recent literature using the same biosusceptometry approach [19]. A schematic illustration of the method and signal analysis is provided in Figure S1.

### 2.4 Sample Collection

After 98 days, the animals were weighed 2 h before being euthanized and received vincristine (Cristália, Itapira, Brazil) (0.5 mg/kg of body weight) intravenously (penile vein) to paralyze cell division in metaphase. After 2 h, the animals were anesthetized with lidocaine (Cristália, Itapira, Brazil) (10 mg/kg) followed by an overdose of thiopental (Cristália, Itapira, Brazil) (150 mg/kg) intraperitoneally. Afterward, they were immobilized so that a celiotomy could be performed. The colon of the animals was collected for evaluation of the experimental model. Then, the jejunum and colon were collected and processed for immunohistochemical analysis and histopathology analysis, respectively.

### 2.5 Analysis of AC, ACF and Histopathology

The total number of AC and ACF was quantified along the entire length of each colonic segment (proximal and distal), which was collected and stained with 1% methylene blue (Sigma‐Aldrich, St. Louis, USA) in 0.1 M PBS pH 7.4 [20]. AC were identified and quantified according to criteria established by Bird et al. (1987) [21] and tabulated as isolated AC and ACF with 2–3, 4–9, or 10 or more AC/focus. The number of AC and ACF/colon was determined using an optical microscope at 40× magnification and analyzed for the following morphological aspects: presence of widened border (if it was slightly raised), oval or slit lumen and intensity of staining relative to adjacent normal colonic crypts. This quantification was performed across the full length of each segment and not normalized per surface area. To account for potential differences in total colonic length between groups, the average colon lengths were measured at the time of sample collection. The total colon length did not significantly differ between groups (C: 18.138 ± 0.94 cm; CR: 17.625 ± 1.026 cm; CQ: 18.213 ± 0.551 cm; CP: 18.188 ± 0.923 cm; CQP: 17.8 ± 0.913 cm).

For histopathological analysis, 1 cm of the proximal and distal colon was collected and then fixed in 10% buffered formalin. The tissue samples were dehydrated in a graded ethanol series (70%, 80%, 95%, and absolute ethanol), diaphonized in xylene, and embedded in paraffin. Sections of 4–5 μm thickness were obtained using a semi‐automatic microtome and mounted on glass slides. The slides were deparaffinized, rehydrated, and stained with hematoxylin (Merck, Darmstadt, Germany) and eosin (Merck, Darmstadt, Germany) for histopathological analysis. They were then examined under light microscopy to identify histopathological alterations consistent with colorectal carcinogenesis.

### 2.6 Study of Enteric Neurons

The jejunum was fixed in Zamboni's fixative (2% paraformaldehyde and 15% picric acid in 0.1 M phosphate buffer, pH 7.4) for 18 h. Segments were longitudinally opened along the mesenteric border and pinned flat over polystyrene plates using wooden fixation pins. No calcium antagonists were used prior to fixation. After fixation, the tissues were washed in 80% alcohol until complete removal of the fixative, dehydrated, diaphonized in xylene, and stored in PBS + 0.08% azide [22]. One square centimeter of the jejunal segments was microdissected and whole mounts of the tunica muscularis and the submucosa were obtained for immunohistochemical staining for the following markers: HuC/D (general population of enteric neurons), nNOS (neuronal nitric oxide synthase), VIP (vasoactive intestinal polypeptide).

To ensure accurate quantification of neuronal density, a correction factor was applied based on the jejunal area of each experimental group. During euthanasia, the length and width of the jejunal segment collected for immunohistochemical analysis were measured using a flexible measuring tape, and the surface area (μm^2^) was estimated by multiplying these dimensions. The control group (C) had a standard jejunum area of 171.0 ± 26.62 μm^2^. The colorectal carcinogenesis group (CR) showed an area of 166.5 ± 23.26 μm^2^, leading to a correction factor of 0.974. Treatment groups receiving microencapsulated quercetin (CQ) and *Bifidobacterium animalis* (CP) had areas of 173.6 ± 20.45 and 183.6 ± 25.61 μm^2^, respectively, resulting in correction factors of 1.015 and 1.074. The combined treatment group (CQP) had a jejunum area measuring 170.3 ± 20.47 μm^2^ with a correction factor of 0.996. The results are expressed as cells per cm^2^ (%) per group [23].

### 2.7 Immunohistochemical Technique

Tissue samples were washed with 0.1 M PBS, with 0.5% Triton (pH 7.4) (Sigma, St. Louis, MO, USA) twice for 10 min; then blocking with PBS 0.1 M was performed, with 2% BSA (bovine serum albumin) (Sigma, St. Louis, MO, USA), 10% donkey serum (Sigma‐Aldrich, St. Louis, USA), 0.5% Triton X‐100 (Sigma‐Aldrich, St. Louis, USA) for 2 h. Following blocking, the tissues were incubated with primary antibodies for 48 h at room temperature. The primary antibodies used included monoclonal mouse anti‐HuC/D at a dilution of 1:400 (Molecular Probes Invitrogen, USA), monoclonal mouse anti‐nNOS at 1:500 (Molecular Probes Invitrogen, USA), and monoclonal rabbit anti‐VIP at 1:250 (Santa Cruz Biotechnology, USA). After incubation with primary antibodies, the tissues underwent three additional washes with PBS for 5 min each. Subsequently, the samples were incubated with the corresponding secondary antibodies for 6 h at room temperature. The secondary antibodies used were Alexa fluor 488 (donkey anti‐mouse) at a dilution of 1:200, Alexa fluor 488 (goat anti‐mouse) at 1:400, and Alexa fluor 488 (goat anti‐rabbit) at 1:400 all produced by Molecular Probes Invitrogen USA. The samples were washed again and mounted with 10% buffered glycerol mounting medium and stored at 4°C [24, 25]. Images were captured using an optical microscope equipped with immunofluorescence filters and a high‐resolution Olympus BX40 camera. Quantitative and morphometric analyses were performed.

### 2.8 Quantification of Immunoreactive Cells (IR) HuC/D, nNOS, VIP

For each group, 32 non‐overlapping images per animal were captured using a 20× objective. Varicosities (VIP) and the populations of myenteric (HuC/D, nNOS) and submucosal (HuC/D, VIP) neurons were quantified for each image. The quantification of the cells present in the images was performed using image analysis by Image Pro Plus 4 software (Media Cybernetics, MD, USA). After density correction, results were expressed as cells per cm^2^ as described by Zanoni et al. [22].

### 2.9 Morphometric Analysis of HuC/D‐IR, nNOS‐IR, and VIP‐IR

For the morphometric analysis of neuronal cell bodies, somatic areas (in μm^2^) of 100 neurons were measured for each animal and for each group. The morphometric evaluation of varicosities (small and frequent neurotransmitter‐containing dilations along the fibers) was performed by measuring areas (in μm^2^) of 250 varicosities per animal from images acquired with a 40× objective. The Image Pro Plus 4 image analysis software was used for the morphometric analysis of all images.

### 2.10 Estimation of Cholinergic Neurons

The estimated cholinergic subpopulation of neurons was obtained by subtracting nNOS^+^ neurons from the total neuronal population (HuC/D‐IR) [9, 26, 27]. The morphometric evaluation was not conducted due to the absence of a valid immunomarker for assessing the population of rat enteric neurons.

### 2.11 Statistical Analyses

Statistical analysis was performed using Statistica (version 7.0, StatSoft) and GraphPad Prism (version 8.0, GraphPad Software). Data were presented as the mean ± standard error of the mean (SEM). A two‐way Analysis of Variance (ANOVA) was employed to analyze the data, with Fisher's post‐hoc test applied to identify specific group differences when ANOVA indicated a significant effect. The significance level for all statistical tests was set at *p <* 0.05. Assumptions of normality and homogeneity of variances were verified using the Shapiro–Wilk test and Levene's test, respectively, prior to performing ANOVA to ensure the validity of the results.

## 3 Results

### 3.1 Gastrointestinal Transit

The mean gastric emptying time (MGET) and mean cecum arrival time (MCAT) were evaluated in all groups. The results show that the experimental group CP had a significantly lower MGET than the C group (*p <* 0.05), while the other experimental groups did not show significant differences. The MCAT values did not differ significantly between the groups. These results, obtained through the magnetic monitoring technique, are presented in Table 1.

**TABLE 1:** Mean gastric emptying time (MGET) and mean cecum arrival time (MCAT) in Wistar rats subjected to colorectal carcinogenesis and treatments with microencapsulated quercetin and *Bifidobacterium animalis* subsp. lactis. C: Control group; CR: Colorectal carcinogenesis group; CQ: CR + microencapsulated quercetin (10 mg/kg); CP: CR + *Bifidobacterium animalis* subsp. lactis (5 × 10^7^ CFU); CQP: CR + combination of both treatments.

Group	MGET	MCAT
C	93.51 ± 10.83	173.74 ± 5.84
CR	86.39 ± 9.17	174.76 ± 3.4
CP	79.77 ± 4.34^#^	175.21 ± 4.62
CQ	87.64 ± 6.89	171.52 ± 12.21
CQP	86.00 ± 5.34	175.17 ± 5.11

*Note:* Data are expressed as mean ± SEM (in minutes). ^#^ *p* < 0.05 versus C. *n*: 8 animals per group.

### 3.2 Validation of the Experimental Model Using 1,2‐Dimethylhydrazine as Chemical Induction of Colonic Cancer

**FIGURE 1:**
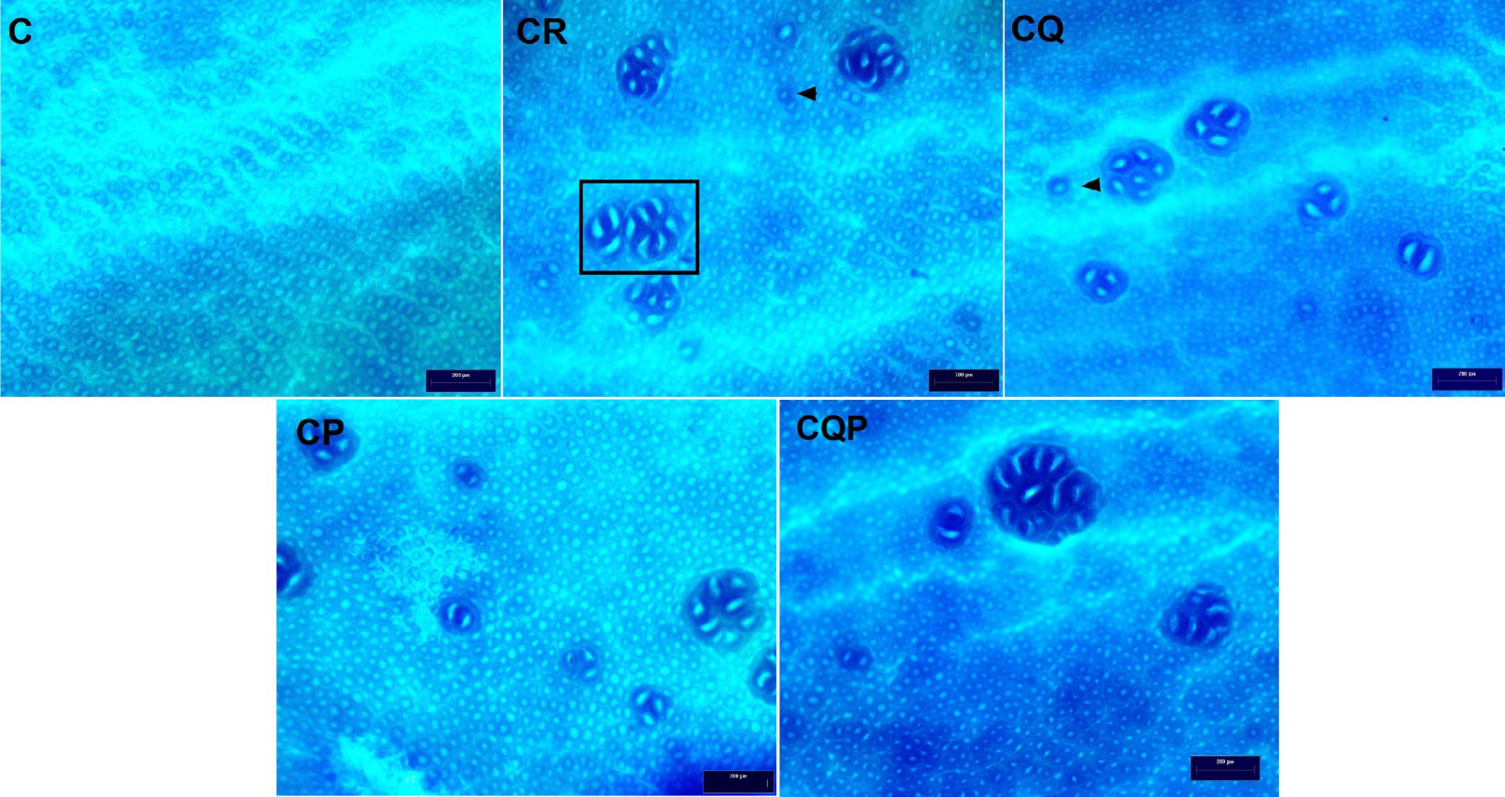
Intraperitoneal administration of 1,2‐dimethylhydrazine (DMH) causes formation of aberrant crypts and foci of aberrant crypts in the colon of Wistar rats. Representative photomicrographs of methylene blue stained colonic samples showing aberrant crypts and foci of aberrant crypts after DMH treatment. C: Control group; CR: Colorectal carcinogenesis group; CQ: CR + microencapsulated quercetin (10 mg/kg); CP: CR + *Bifidobacterium animalis* subsp. lactis (5 × 10^7^ CFU); CQP: CR + combination of both treatments. Black arrows indicate aberrant crypts, and the insert shows two aberrant crypt foci, containing 3 and 6 aberrant crypts, respectively. Magnification: 4×. Scale bar: 200 μm.

In the CR, CQ, CP, and CQP groups, the presence of AC and ACF was confirmed in both proximal and distal colonic segments of all animals (*p <* 0.05 vs. C; Figure 1; Table 2). No ACF containing ≥ 10 crypts was observed in the proximal colon of animals in the CQ group.

**TABLE 2:** Quantification of aberrant crypts (AC) and aberrant crypt foci (ACF) in the proximal and distal colon of Wistar rats subjected to colorectal carcinogenesis and treatments with microencapsulated quercetin and *Bifidobacterium animalis* subsp. lactis. C: Control group; CR: Colorectal carcinogenesis group; CQ: CR + microencapsulated quercetin (10 mg/kg); CP: CR + *Bifidobacterium animalis* subsp. lactis (5 × 10^7^ CFU); CQP: CR + combination of both treatments.

Group	AC		ACF					
	Proximal	Distal	Proximal			Distal		
			2–3	4–9	≥ 10	2–3	4–9	≥ 10
C	0 ± 0	0 ± 0	0 ± 0	0 ± 0	0 ± 0	0 ± 0	0 ± 0	0 ± 0
CR	31.6 ± 18.7^#^	13.6 ± 14.3^#^	49.1 ± 40.1^#^	19.1 ± 18.3^#^	0.1 ± 0.4^#^	42.4 ± 21.2^#^	62.8 ± 30.1^#^	8.3 ± 9.0^#^
CQ	20.0 ± 14.1^#^	16.0 ± 9.8^#^	32.9 ± 28.1^#^	13.9 ± 15.2^#^	0 ± 0	45.4 ± 16.1^#^	70.5 ± 20.5^#^	8.4 ± 5.8^#^
CP	31.1 ± 15.2^#^	21.3 ± 12.9^#^	61.4 ± 18.4^#^	25.4 ± 16.8^#^	0.1 ± 0.4^#^	67.1 ± 32.1^#^	83.9 ± 35.2^#^	11 ± 12.9^#^
CQP	34.5 ± 32.1^#^	12.6 ± 7.2^#^	53.9 ± 46.2^#^	22 ± 28.3^#^	0.1 ± 0.4^#^	58.5 ± 24.5^#^	98.6 ± 53.7^#^	12.3 ± 13.2^#^

*Note:* Data are expressed as mean ± SEM. ^#^ *p* < 0.05 versus control group. *n*: 8 animals per group.

The number of AC in the proximal region of CQ, CP, and CQP was like the CR group (*p* > 0.05). ACF with 4–9 crypts was highly prevalent throughout the distal colon (Figure 1; Table 2). No statistically significant differences were observed between the treated groups (CQ, CP, and CQP) and the CR group.

**FIGURE 2:**
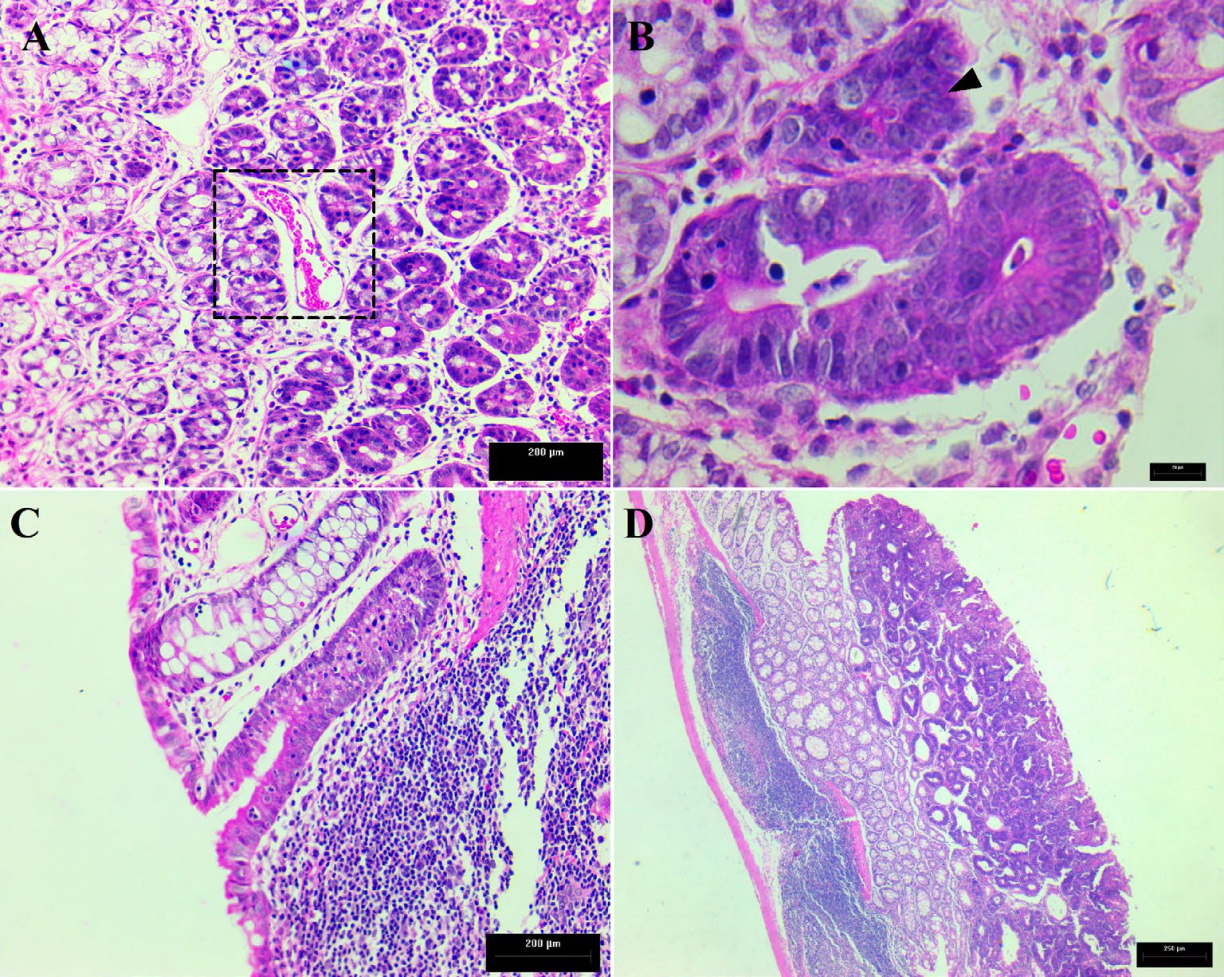
Histopathological evaluation of HE‐stained rat colonic microscopic sections after DMH treatment showing. (A) Hyperplasia of the colonic epithelium and vascular congestion in the CQ group (Scale bar: 200 μm; Magnification: 200×). (B) Dysplastic epithelium with nuclear atypia in the CP group (Scale bar: 20 μm; Magnification: 400×). (C) Lymphocytic inflammatory infiltrates and Peyer's patches in the CQP group (Scale bar: 200 μm; Magnification: 200×). (D) Polypoid formation with severe dysplasia and architectural disorganization in the CR group (Scale bar: 250 μm; Magnification: 40×). CR: Colorectal carcinogenesis group; CQ: CR + microencapsulated quercetin (10 mg/kg); CP: CR + *Bifidobacterium animalis* subsp. lactis (5 × 10^7^ CFU); CQP: CR + combination of both treatments. These findings are representative of the respective induced groups.

Dysplastic lesions were observed in all specimens in the cancer‐bearing groups. In addition, diffuse Peyer's patches along with lymphocytic infiltrates and congestion in tissue vessels were evidenced in the distal colon. An adenomatous polypoid formation was observed, showing areas with severe dysplasia, architectural disorganization, and atypical nuclear morphology (Figure 2).

### 3.3 Neuronal Density and Morphometry

**FIGURE 3:**
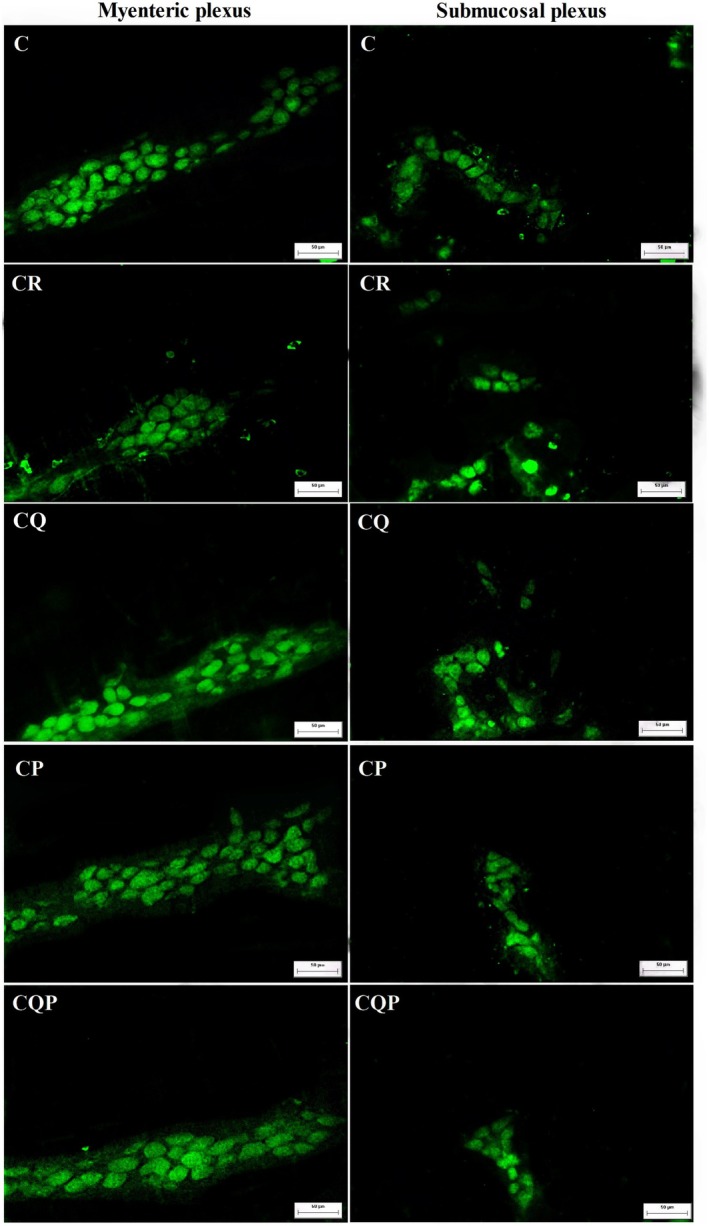
HuC/D+ neurons of the myenteric and submucosal plexus in the jejunum of Wistar rats chemically induced colorectal carcinogenesis. Representative photomicrographs of the myenteric and submucosal plexus of the jejunum of Wistar rats submitted to immunohistochemistry for HuC/D. C: Control group; CR: Colorectal carcinogenesis group; CQ: CR + microencapsulated quercetin (10 mg/kg); CP: CR + *Bifidobacterium animalis* subsp. lactis (5 × 10^7^ CFU); CQP: CR + combination of both treatments. Magnification: 200×. Scale bar: 50 μm.

**FIGURE 4:**
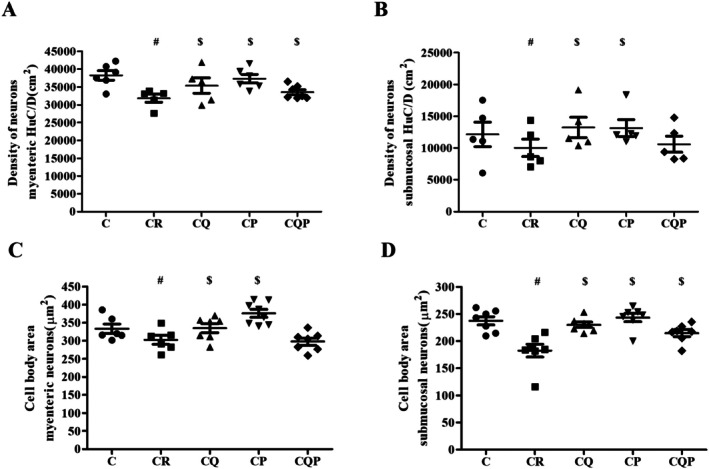
Colorectal carcinogenesis reduced the density and size of HuC/D+ neurons in the myenteric and submucosal plexus. (A) Density and (C) size of HuC/D‐immunoreactive (HuC/D‐IR) neurons in the myenteric plexus. (B) Density and (D) size of HuC/D‐IR neurons in the submucosal plexus. C: Control group; CR: Colorectal carcinogenesis group; CQ: CR + microencapsulated quercetin (10 mg/kg); CP: CR + *Bifidobacterium animalis* subsp. lactis (5 × 10^7^ CFU); CQP: CR + combination of both treatments. ^#^ *p* < 0.05 versus Control group. ^$^ *p* < 0.05 versus Colorectal Carcinogenesis (CR) group. Data are expressed as mean ± SEM. *n*: 6 animals per group.

To perform quantitative analyses in terms of neuronal density and morphology in the ENS of the control group and the 4 treated groups, immunostaining with the general neuronal marker HuC/D was performed (Figure 3). In HuC/D‐IR neurons, there was a density reduction of 21.92% and 16.78% in the myenteric and submucosal plexus, respectively (CR vs. C; *p <* 0.05; Figure 4A,B). In the myenteric plexus of the CQ and CP groups, there was an increase of 11.09% and 13.34%, respectively, in neuronal density (CQ and CP vs. CR; *p <* 0.05; Figure 4A). In the submucosal plexus, neuronal density increased by 31.92% in CQ and 25.31% in CP (*p <* 0.05; Figure 4B).

There was a reduction in the cell body area of myenteric (9.22%) and submucosal (23.20%) neurons (CR vs. C; *p <* 0.05; Figure 4C,D). In the myenteric plexus, the neuronal area increased by 10.66% in the CQ and 24.45% in the CP (vs. CR; *p <* 0.05; Figure 4C). In the CQ, CP, and CQP groups, an increase of 26.08%, 34.26%, and 17.89% was observed in the submucosal plexus, respectively, in the bodies of neurons (vs. CR; *p <* 0.05; Figure 4D).

### 3.4 Varicosities and VIP‐IR Subpopulation in the Myenteric and Submucosal Plexus

**FIGURE 5:**
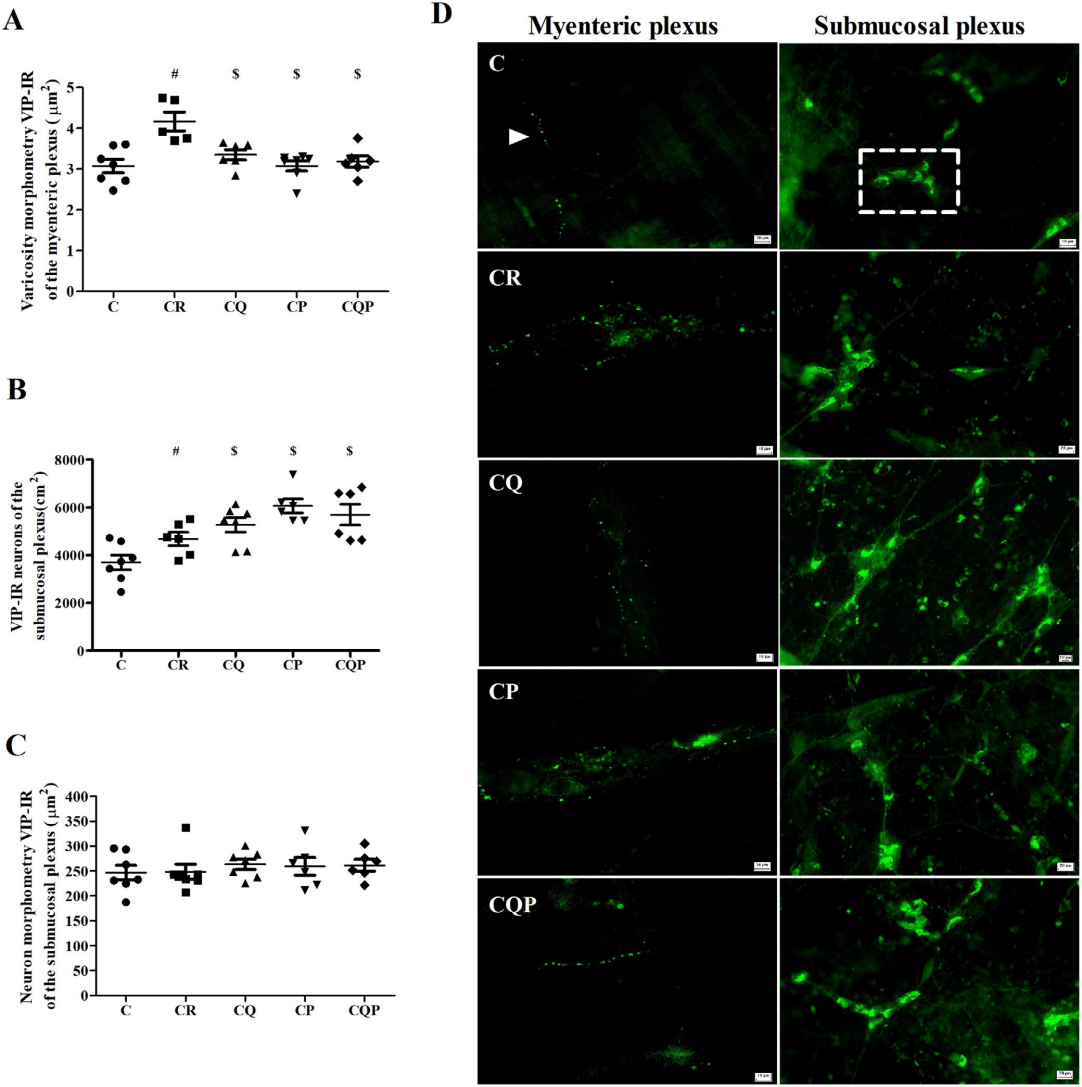
Colorectal carcinogenesis increases VIP expression in the myenteric and submucosal plexuses, which is modulated by treatment with microencapsulated quercetin and *Bifidobacterium animalis* . (A) Morphometric analysis of VIP+ varicosities in the myenteric plexus. (B) Density and (C) size of VIP‐immunoreactive (VIP‐IR) neurons in the submucosal plexus. (D) Representative immunohistochemical photomicrographs of VIP‐IR neurons in the myenteric and submucosal plexuses from each experimental group. A white arrow indicates VIP‐IR varicosities, and the insert highlights a submucosal ganglion containing five VIP‐IR neurons. C: Control group; CR: Colorectal carcinogenesis group; CQ: CR + microencapsulated quercetin (10 mg/kg); CP: CR + *Bifidobacterium animalis* subsp. lactis (5 × 10^7^ CFU); CQP: CR + combination of both treatments. ^#^ *p* < 0.05 versus Control group. ^$^ *p* < 0.05 versus Colorectal Carcinogenesis (CR) group. Magnification: 200×. Scale bar: 10 μm. *n*: 6 animals per group.

To perform quantitative analyses of VIPergic expression in the ENS of the control and treated groups, immunostaining for VIP was performed. Morphometric analysis of varicosities was conducted in the myenteric plexus, while neuronal density and size were assessed in the submucosal plexus (Figure 5D). The VIPergic subpopulation, in the myenteric plexus, when analyzing the area of neuronal varicosities, we found an increase of 35.55% in the CR group (vs. C; *p <* 0.05; Figure 5A). On the other hand, there was a reduction of 19.64%, 26.14% and 23.49% in CQ, CP and CQP, respectively (vs. CR; *p <* 0.05; Figure 5A). In the submucosal plexus, VIPergic neuronal density increased by 26.44% (CR vs. C; *p <* 0.05). Regarding the VIPergic subpopulation in the myenteric plexus, we demonstrated an increase of 12.68% in CQ, 29.65% in CP and 21.79% in CQP in VIP‐IR neuronal submucous density (vs. CR; *p <* 0.05, Figure 5B). The size of neurons in the VIPergic subpopulation remained unchanged (CR vs. C; CQ, CP and CQP vs CR; *p >* 0.05; Figure 5C).

### 3.5 Neuronal Nitrergic Density (nNOS‐IR) in the Myenteric Plexus

**FIGURE 6:**
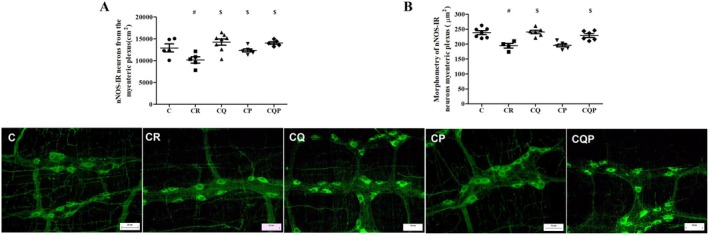
Administration of microencapsulated quercetin and *Bifidobacterium animalis* preserved the density and size of myenteric nNOS^+^ neurons in the jejunum in rats with DMH‐induced colorectal carcinogenesis. (A) Size and (B) density of nNOS‐immunoreactive (nNOS‐IR) neurons in the myenteric plexus. (C) Representative immunohistochemical photomicrographs of nNOS‐IR neurons in the myenteric plexus from each experimental group (Scale bar: 50 μm). C: Control group; CR: Colorectal carcinogenesis group; CQ: CR + microencapsulated quercetin (10 mg/kg); CP: CR + *Bifidobacterium animalis* subsp. lactis (5 × 10^7^ CFU); CQP: CR + combination of both treatments. ^#^ *p* < 0.05 versus Control group. ^$^ *p* < 0.05 versus Colorectal Carcinogenesis (CR) group. Magnification: 200×. *n*: 6 animals per group.

To analyze the density and morphology of nitrergic neurons in the myenteric plexus, we performed immunostaining for nNOS‐IR (Figure 6C). In the subpopulation of nitrergic neurons (nNOS‐IR) in the myenteric plexus, we observed a 21% reduction in CR neuronal density (CR vs. C; *p <* 0.05; Figure 6A). Additionally, there was an increase in CQ, CP, and CQP by 40.42%, 21.51%, and 38.35% respectively, compared to CR (*p <* 0.05; Figure 6A). Morphometric analysis revealed a 19.22% decrease in CR (vs, C; *p <* 0.05; Figure 6B). Moreover, the CQ and CQP groups exhibited a 23.51% and 17.46% increase respectively, compared to CR (*p <* 0.05; Figure 6B).

### 3.6 Estimated Cholinergic Population

The estimated density of the cholinergic subpopulation showed no significant difference between the CR and control (C) groups (*p >* 0.05). No significant differences were observed in cholinergic neuronal density between the CR group and the CQ, CP, and CQP groups (*p >* 0.05). The percentage changes in cholinergic neuronal density were an increase of 0.79% and 6.53% in the CQ and CP groups, respectively, and a reduction of 19.82% in the CQP group, compared to the CR group.

## 4 Discussion

Colorectal carcinogenesis was induced using 1,2‐dimethylhydrazine (DMH), a highly specific and indirect carcinogen that both initiates and promotes CRC [28]. DMH and its metabolite azoxymethane are procarcinogens that require metabolic activation to form DNA‐reactive products, acting as alkylating agents that initiate their mutagenic activity through methylation of guanine at the N‐7 position in DNA [29]. In our study, exposure to DMH was able to produce tumor lesions in 100% of the animals in the 4 treated groups: (a) a group receiving only DMH treatment (CR), (b) a group receiving DMH treatment + administration of quercetin (CQ), (c) a group receiving DMH + administration of a probiotic ( *Bifidobacterium animalis* subtype lactis) (CP), and (d) a group receiving DMH + administration of both quercetin and the probiotic. (CQP).

Consistent with our findings, the study by Lin et al. [30] demonstrated that *Bifidobacterium animalis* subtype *lactis*, although effective in reducing oxidative stress, was not able to reduce the number of ACF induced by DMH in Wistar rats. In our study, neither microencapsulated quercetin nor *B. animalis* , alone or in combination, significantly altered the formation of aberrant crypts, suggesting that these compounds may be more effective at modulating downstream events of colorectal carcinogenesis.

Our experimental model confirmed that colorectal carcinogenesis induces not only colonic mucosal alterations—characterized by polypoid formations, severe dysplasia, nuclear atypia, and architectural disorganization—but also significant changes in the small intestine, expanding upon recent studies that focused on the large intestine [5, 31, 32]. Indeed, we observed a reduction in the density and size of the general population of neurons within the small intestine, as evidenced by the decreased expression of both submucous and myenteric HuC/D‐IR jejunal neurons, which agrees with some recent studies. In the small intestine, the ENS plays a crucial role in controlling nutrient absorption, secretions, motility, and blood flow [6]. However, the relationship between colorectal carcinogenesis and enteric innervation components in the small intestine has not been described in the literature. Our results are also consistent with previous data from our group, using rats with Walker 256 tumor, demonstrating a similar reduction in neuronal density in the ENS of the jejunum of these animals and a decreased VIPergic expression [23].

Although the CR group exhibited enteric neuronal loss, gastrointestinal motility remained unchanged due to the plasticity of the ENS, which enables compensatory mechanisms such as increased neurotransmitter release and enhanced activity of interstitial cells of Cajal to preserve function [6, 31]. However, as the disease progresses, inflammation and oxidative stress exacerbate ENS neuronal loss, impairing motility regulation and potentially leading to delayed dysfunction [33, 34].

In the CQP group, the absence of an additive effect from the combination therapy is probably due to the antimicrobial activity of quercetin, which has been shown to inhibit certain *Bifidobacterium* strains [35, 36]. This suggests that quercetin might modulate the gut microbiota composition, potentially affecting the probiotic functions of *Bifidobacterium*, and may exacerbate ENS neuronal loss.

We showed an increase in the size of varicosities and the density of VIPergic neurons in rats from the CR group. VIP is a peptide that has already been described as an important tumor growth factor; its VPAC1 receptor is overexpressed in several types of cancer, such as colon, pancreas, and lung. Furthermore, VPAC1, is identified as an important possible therapeutic target in the treatment of CRC [37]. Changes in VIP expression have the potential to alter motility and nutrient absorption in the small intestine [38, 39, 40] which can result in intestinal obstruction, a common complication in gastrointestinal tract neoplasms [41].

In contrast to the CR group, treatment with microencapsulated quercetin, *B. animalis* , or their combination significantly reduced VIPergic varicosities in the myenteric plexus, suggesting a protective neuromodulatory effect. This modulation is particularly relevant given that elevated VIP expression has been associated with more aggressive CRC phenotypes. However, in the submucosal plexus, this effect was not observed in the groups treated with the probiotic, which may be explained by the action of microbial metabolites—especially short‐chain fatty acids (SCFAs) such as butyrate, propionate, and acetate—that can act as epigenetic modulators and enhance the expression of neurotrophic peptides like VIP [42, 43]. Despite these findings, the precise role of VIP in CRC progression remains controversial and warrants further investigation [44, 45].

Sitohy and El‐Salhy [46] reported a decrease in both the submucous and myenteric nitrergic subpopulations in the colon of animals exposed to DMH. Similarly, we observed a reduction in the size and density of nNOS‐IR neurons in the myenteric plexus in animals from the CR group. Nitric oxide induces vascular relaxation, and NOS inhibitors are selectively used to reduce blood flow in angiogenesis that is associated with tumor development [47, 48]. On the other hand, in the jejunum of cachectic rats with Walker‐256 tumor, an increase in nNOS was associated with the preservation of interstitial cells of Cajal [23]. The administration of quercetin was able to preserve the size and population density of nNOS‐IR neurons; similar effects were described in the nitrergic population of diabetic rats [9].

Interestingly, despite the changes in VIP and nNOS levels, we did not observe any significant alterations in intestinal motility in these groups. This may be due to compensatory mechanisms in the ENS, such as changes in other neurotransmitters or the activation of other signaling pathways, that maintain overall gastrointestinal motility despite changes in individual neurotransmitters [49]. Acetylcholine has been shown to promote intestinal smooth muscle contraction and enhance gastrointestinal motility [50].

In the CP group, the administration of *B. animalis* led to a significant reduction in mean gastric emptying time (MGET). Interestingly, this reduction did not correspond to a significant change in mean cecum arrival time (MCAT). The changes in gastric emptying time following *B. animalis* administration may be attributed to its modulatory effects on gut microbiota and enteric neurotransmission. Probiotics have been shown to regulate gastrointestinal motility through their interaction with gut microbiota‐derived metabolites, such as SCFAs, which can influence the release of motility‐related hormones like glucagon‐like peptide‐1 (GLP‐1) and peptide YY (PYY) [51, 52]. The selective effect of *B. animalis* on gastric emptying, rather than overall transit time, suggests a localized modulation of gastric motility rather than a systemic restoration of ENS function.

An important factor for tumor development, oxidative stress, is characterized by an imbalance between oxidizing agents and endogenous antioxidant mechanisms [53]. To strengthen the antioxidant systems and thereby minimize oxidative stress, we use microencapsulated quercetin. Doses of 150 mg/kg of quercetin are known to reduce inflammation and increase antioxidant defenses in arthritic rats [54], however studies show an increase in the risk of toxicity caused by quercetin from 100 mg/kg [55, 56]. In addition, low doses of quercetin have reduced antioxidant potential [8, 57]. A modified release system, such as nanoparticles and microcapsules, makes it possible to increase the bioavailability of quercetin, allowing us to use lower doses [8, 9, 58].

*Bifidobacterium* bacteria are known to be beneficial for the host's health, including competitive exclusion of pathogens, modulation of the immune system, and degradation of carbohydrates derived from the diet [59]. In addition, in chemically induced colorectal carcinogenesis, *B. animalis* has been shown to inhibit oxidative stress [30]. Our data demonstrate the protective effect of administering microencapsulated quercetin in both plexuses. Another study has also shown the neuroprotective effect of microencapsulated quercetin in the ENS in a model of diabetes, using a concentration of 10 mg/kg/day, which is the same dosage used in our study [9].

Despite the promising findings, some limitations should be considered in the interpretation of our results. First, the analysis of neuronal subpopulations was conducted using single immunostaining, without double‐labeling techniques, which limit the precise identification of neuronal phenotypes and possible co‐expression patterns. Second, only male rats were included in the experimental design to avoid hormonal fluctuations, which restrict the generalizability of the findings to both sexes. Finally, while our results demonstrate short‐term protective effects of individual treatments, long‐term outcomes and dose–response relationships were not assessed and warrant further investigation.

In this study, while quercetin and *B. animalis* individually exhibited neuroprotective effects, their co‐administration did not yield the same benefit. This may be due to metabolic interactions, where quercetin's antioxidant and anti‐inflammatory actions could have altered the intestinal microbiota, compromising the probiotic's viability and function. Additionally, competition for metabolic substrates in the intestinal lumen may have reduced the efficacy of the combined treatment. These findings align with studies showing that flavonoids can modulate gut microbiota composition and activity, influencing neuroinflammation and intestinal barrier integrity [60, 61]. Importantly, this is the first study to demonstrate that colorectal carcinogenesis induces enteric neuronal loss in the small intestine, highlighting the need for further research on ENS preservation beyond the colon.

## 5 Conclusion

This study demonstrated the impact of CRC on the ENS in the jejunum, showing reductions in neuronal density and size. Importantly, it demonstrated the protective effects of microencapsulated quercetin and *B. animalis* subtype lactis but not the combination.

Microencapsulated quercetin preserved enteric neurons and reduced VIPergic varicosities, maybe due to its antioxidant and anti‐inflammatory properties. *B. animalis* subtype lactis improved antioxidant defenses and preserved neuronal populations, also reducing mean gastric emptying time.

Interestingly, when quercetin and *B. animalis* were administered together, their combined effect did not enhance the observed benefits compared to each treatment individually. In some cases, the combined treatment appeared to counteract the effects observed with the single treatments, suggesting a potential interaction between them that requires further investigation.

Microencapsulated quercetin and *B. animalis* subtype lactis show potential as adjunct therapies in CRC management, but there is a need for further research to optimize their use and explore their mechanisms of action.

## Author Contributions

Lucas Casagrande, Carla Cristina de Oliveira Bernardo, Juliana Vanessa Colombo Martins Perles, Sabrina Silva Sestak, Tânia Cristina Alexandrino Becker and Jacqueline Nelisis Zanoni designed and coordinated the study; Lucas Casagrande, Carla Cristina de Oliveira Bernardo, Sabrina Silva Sestak, Maysa Pacheco Alvarez da Silva, Cesar Agostinho Ferreira, Tânia Cristina Alexandrino Becker, Erick Guilherme Stoppa and José Ricardo de Arruma Miranda performed the experiments, acquired, and analyzed data; Lucas Casagrande, Carla Cristina de Oliveira Bernardo, Sabrina Silva Sestak, Maysa Pacheco Alvarez da Silva, Cesar Agostinho Ferreira, Tânia Cristina Alexandrino Becker, Erick Guilherme Stoppa, Waldiceu Aparecido Verri, José Ricardo de Arruma Miranda, Jean‐Pierre Timmermans, Juliana Vanessa Colombo Martins Perles, and Jacqueline Nelisis Zanoni interpreted the data; Lucas Casagrande, Juliana Vanessa Colombo Martins Perles, Jean‐Pierre Timmermans, Jacqueline Nelisis Zanoni, and wrote the manuscript; all authors approved the final version of the article.

## Conflicts of Interest

The authors declare no conflicts of interest.

## Supporting information


**Appendix S1:** nmo70190‐sup‐0001‐AppendixS1.docx.

## Data Availability

The data that support the findings of this study are available from the corresponding author upon reasonable request.
