# Supplementary material for: Microencapsulated Quercetin and Bifidobacterium animalis Independently Preserve Jejunal Enteric Neurons During Colorectal Carcinogenesis
Source: Neurogastroenterol Motil. 2025 Oct 29;38(1):e70190. doi: 10.1111/nmo.70190 (PMC12815002; doi:10.1111/nmo.70190)
Supplement: Supplementary file 1 — Appendix S1: nmo70190‐sup‐0001‐AppendixS1.docx. [file NMO-38-e70190-s001.docx]

# **SUPPLEMENTARY FIGURE 1 – SCHEMATIC REPRESENTATION OF THE MAGNETIC BIOSUSCEPTOMETRY METHOD**


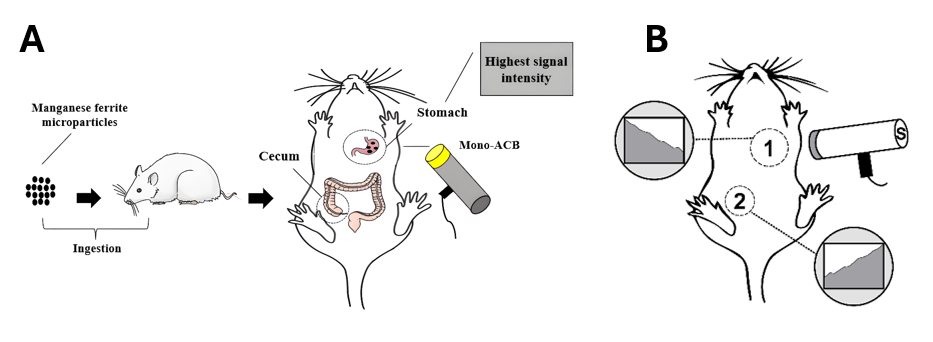


**Supplementary Figure 1. Schematic representation of the magnetic biosusceptometry method used to evaluate gastrointestinal transit.** In (A) Tracer induction and sensor positioning: Shows the process of manganese ferrite microparticles ingestion by the rat, which act as a magnetic tracer. An external sensor (Mono-ACB) is positioned over the gastric region to detect the magnetic signal intensity. The highest signal intensity is initially recorded in the stomach, indicating the presence of the microparticles. In (B) Measurement setup and signal curves: Presents the positioning of the external sensors over the rat, with one sensor over the gastric region (1) and another over the cecum (2). The inserted graphs demonstrate the signal intensity curves obtained at each location: in the gastric region (1), a progressive decrease in signal intensity is observed over time, indicating emptying; in the cecum region (2), there is a progressive increase in signal intensity as the magnetic microparticles reach this area. The mean gastric emptying time (MGET) and mean caecum arrival time (MCAT) are calculated based on the first temporal statistical moment of these curves.
